# Behind Brain Metastases Formation: Cellular and Molecular Alterations and Blood–Brain Barrier Disruption

**DOI:** 10.3390/ijms22137057

**Published:** 2021-06-30

**Authors:** Joana Godinho-Pereira, Ana Rita Garcia, Inês Figueira, Rui Malhó, Maria Alexandra Brito

**Affiliations:** 1 iMed.ULisboa—Research Institute for Medicines, Faculty of Pharmacy, Universidade de Lisboa, Av. Prof. Gama Pinto, 1649-003 Lisbon, Portugal; joanagpereira@ff.ulisboa.pt (J.G.-P.); arcgarcia@campus.ul.pt (A.R.G.); ifigueira@farm-id.pt (I.F.); 2 Department of Pharmaceutical Sciences and Medicines, Faculty of Pharmacy, Universidade de Lisboa, Av. Prof. Gama Pinto, 1649-003 Lisbon, Portugal; 3 Farm-ID—Faculty of Pharmacy Association for Research and Development, Av. Prof. Gama Pinto, 1649-003 Lisbon, Portugal; 4 BioISI—Biosystems and Integrative Sciences Institute, Faculty of Sciences, Universidade de Lisboa, Campo Grande 016, 1749-016 Lisbon, Portugal; rmmalho@fc.ul.pt

**Keywords:** blood–brain barrier, breast cancer brain metastases, extravasation, paracellular and transcellular migration, adhesion, cellular communication

## Abstract

Breast cancer (BC) brain metastases is a life-threatening condition to which accounts the poor understanding of BC cells’ (BCCs) extravasation into the brain, precluding the development of preventive strategies. Thus, we aimed to unravel the players involved in the interaction between BCCs and blood–brain barrier (BBB) endothelial cells underlying BBB alterations and the transendothelial migration of malignant cells. We used brain microvascular endothelial cells (BMECs) as a BBB in vitro model, under conditions mimicking shear stress to improve in vivo-like BBB features. Mixed cultures were performed by the addition of fluorescently labelled BCCs to distinguish individual cell populations. BCC–BMEC interaction compromised BBB integrity, as revealed by junctional proteins (β-catenin and zonula occludens-1) disruption and caveolae (caveolin-1) increase, reflecting paracellular and transcellular hyperpermeability, respectively. Both BMECs and BCCs presented alterations in the expression pattern of connexin 43, suggesting the involvement of the gap junction protein. Myosin light chain kinase and phosphorylated myosin light chain were upregulated, revealing the involvement of the endothelial cytoskeleton in the extravasation process. β4-Integrin and focal adhesion kinase were colocalised in malignant cells, reflecting molecular interaction. Moreover, BCCs exhibited invadopodia, attesting migratory properties. Collectively, hub players involved in BC brain metastases formation were unveiled, disclosing possible therapeutic targets for metastases prevention.

## 1. Introduction

Breast cancer (BC) represents the most commonly diagnosed and leading cause of neoplastic disease in woman, with more than 2.2 million new cases and 684,996 deaths in 2020 [1]. Advances in the early detection and treatment of the primary tumour have led to an increase in cancer survivors [2], rendering the largely incurable metastatic recurrence the foremost concern for cancer patients [3]. Indeed, circa 15% of BC patients present brain metastases [4]. BC brain metastases particularly occur in triple negative BC, and represent a poor prognosis condition, with a low rate of patient survival [5,6].

It is known that BC brain metastases formation occurs through a process named the metastatic cascade [7] that involves the exit of BC cells (BCCs) from the mammary ducts (invasion), the entrance of malignant cells in circulation (intravasation), their survival in the bloodstream and their arrival to the target organ, where they can transmigrate (extravasation) through the blood–brain barrier (BBB) and finally colonise the brain [7]. The extravasation process comprises three major sequential steps, rolling, adhesion and transendothelial migration (TEM) [8], involving several receptors and ligands such as selectins, integrins and members of the immunoglobulin superfamily of cell adhesion molecules [9,10].

Adhesion molecules and signalling proteins, such as focal adhesion kinase (FAK), can have a determinant role in the adhesion process of both endothelial and BCCs [11,12]. FAK’s importance towards BCC migration and invasion mechanisms [13], as well as in endothelial cells’ (ECs) permeability upon contact with BCCs [14,15] have been demonstrated. However, FAK’s function in brain microvascular EC (BMEC)–BCC interaction during extravasation remains under-investigated.

In order to extravasate into the brain parenchyma, BCCs must surpass the BBB [16]. The anatomical basis of the BBB is formed by BMECs, which are characterised by the presence of strong junctional complexes, mostly formed by tight and adherens junctions (TJs and AJs, respectively), which account for the restricted BBB permeability [16]. The mechanical forces promoted by the blood flow to which BMECs are subjected, known as the fluidic shear stress (SS), lead to cytoskeleton rearrangements (i.e., cell elongation and alignment), barrier tightness and restricted permeability as a consequence of TJs’ and AJs’ increased expression [17,18]. Despite the obstacle posed by the BBB, BCCs are able to cross it, as revealed by the establishment of brain metastases [19].

Although the paracellular route seems to be the most commonly used by tumour cells [20,21,22], the transcellular one has also been reported as a possible way of BCCs to transpose the BBB [23,24], with a better understanding of the pathway used by the BCCs during TEM being imperative. In fact, the activation of endothelial protein kinases such as myosin light chain kinase (MLCK), which controls myosin light chain (MLC) phosphorylation, prompting stress fibre formation [25] and cytoskeleton contractility [26], is thought to play a key role in tumour cell migration [27]. Moreover, endothelial MLCK was already identified as a player in transcellular BCC intravasation [28]; however, its role in extravasation has been underexplored.

Vesicular transcytosis is intimately associated with transcellular transmigration, where caveolin-1 (cav-1) is described to play a role, particularly in BMECs, regulating BBB permeability [29]. Interestingly, cav-1 increased expression in the microvasculature along in vivo BC brain metastasis formation has recently been reported by our team [19], suggesting an increase in transcellular permeability. As far as cancer is concerned, cav-1 has been described to bear a role in BCC metastatic mechanisms, though conflicting information has been provided, with reports that its overexpression has either pro- or anti-metastatic properties [30,31]. In fact, cav-1 upregulation was associated with resistance to anoikis, a programmed type of apoptosis resulting from the loss of cell–matrix adhesion [32], in line with its pro-metastatic activity, while decreasing the proliferation and migration of the BCCs with brain tropism, 231-BR [33], consistent with cav-1’s anti-metastatic role.

Cell communication is paramount to extravasation and tumour formation. Gap junction proteins, such as connexin 43 (Cx43), allow cellular communication by the formation of intercellular channels among adjacent cells, impacting cell–cell adhesion, migration, proliferation, and permeability [34,35,36]. Endothelial Cx43, in particular, was described to have a role in cell communication and BBB function [35]. However, authors are not in agreement on Cx43’s role in metastases development, with some describing it as a tumour suppressor and others as a key molecule involved in tumourigenesis [34,37]. Nevertheless, Cx43 appears to play a role in cancer cell transmigration as it localises in the interface between endothelial and BCCs during this process, being involved in BCC migration induction [38]. Accordingly, along in vivo BC brain metastasis formation, intercellular communication via Cx43 was indicated by the protein expression among BCCs, and between BCCs and BMECs [19].

In this work, we aimed to unravel key players involved along the interaction between triple negative BCCs and BMECs, which underlay BBB alterations and transendothelial migration mechanisms of malignant cells towards the brain. We took advantage of an in-house improved BBB in vitro model, which better mimics the in vivo features by encompassing the effect of SS [17], where fluorescently labelled triple negative BCCs were added. Using this cell model of BC brain metastasis formation, we provide evidence regarding signalling molecules involved in BCCs’ migratory phenotype, as well as in their adhesion and TEM across the BBB endothelium, essential for BCC extravasation and BC brain metastasis formation. Along BCC–BMEC interaction, Cx43 delocalisation was observed, pointing to a role of the gap junction protein. Endothelial vesicular transcytosis activation via cav-1, cytoskeleton-associated kinase expression with concomitant cytoskeleton contractility and rearrangements, and junctional complex disturbances associated with endothelial monolayer impairment were observed, indicating both paracellular and transcellular alterations during TEM, as well as BBB disruption. Collectively, with this work, we disclosed the molecular players and mechanisms involved in BCC–BMEC interaction, possible targets for modulation to prevent the BCCs’ extravasation and BC brain metastases development, either by BBB properties’ improvement or towards the decrease in BCCs’ adhesion and migratory properties.

## 2. Results

### 2.1. SS Promotes In Vivo-Like BBB Properties

**Figure 1:**
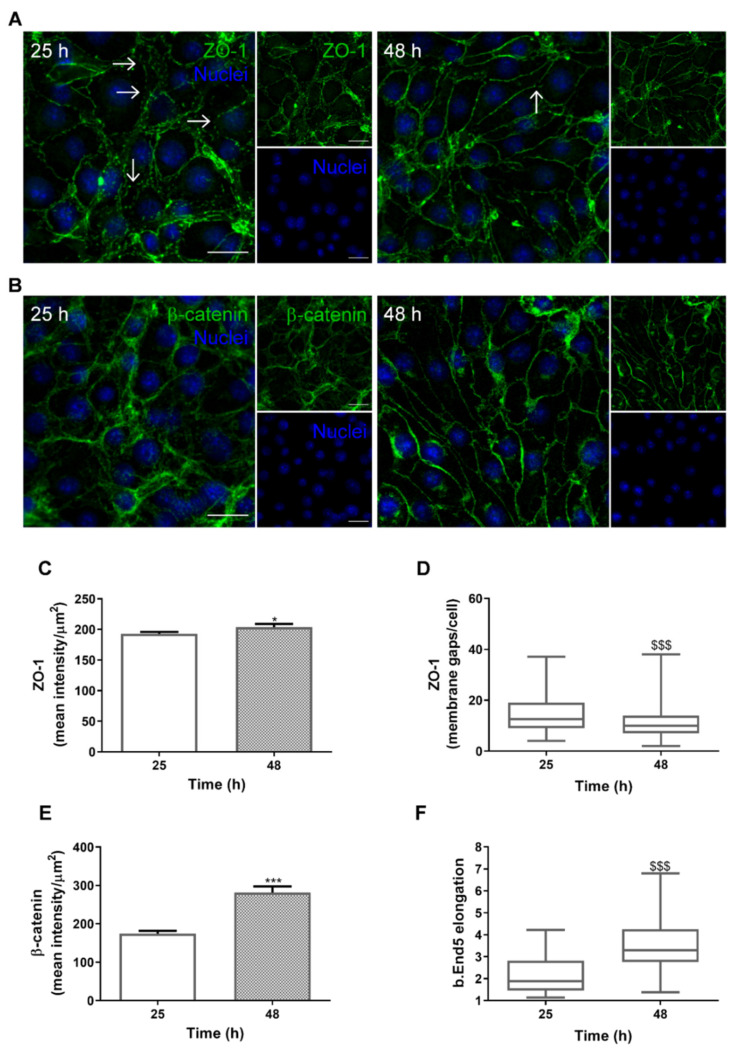
Shear stress (SS) promotes brain microvascular endothelial properties. Confluent monolayers of the brain endothelioma cell line b.End5 were exposed to physiological laminar non-pulsatile SS for 25 and 48 h. SS effects were evaluated by immunofluorescence analysis of the tight and adherens junction proteins (**A**) zonula occludens (ZO)-1 and (**B**) β-catenin, respectively, which showed a delocalisation of the proteins towards the cell membrane and a decrease in membrane gaps (white arrows). Hoechst 33342 was used as counterstaining for nuclei (blue). Scale bar: 20 µm. Semi-quantitative analysis of ZO-1 expression showed (**C**) a slight increase in staining intensity, from 25 to 48 h, and a (**D**) marked decrease in cell membrane gaps, while β-catenin presented (**E**) a notorious increase in fluorescence intensity, and (**F**) a clear cellular elongation. Data are given as means ± SEM (*n* = 3, 10 fields/condition). A Student’s *t*-test for mean intensity and a Mann–Whitney test for membrane gaps and cell elongation were used to evaluate the significant differences, where * *p* < 0.05 and *** *p* < 0.001 or ^$$$^ *p* < 0.001 denote differences between the indicated timepoints.

Our previous study demonstrated that monolayers of the brain endothelioma cell line b.End5 exposed to orbital rotation to mimic physiological values of SS (1.5 dyn/cm^2^) present an increased expression of junctional complexes and an elongated and aligned morphology, in line with the in vivo properties of BMECs [17]. To characterise the behaviour of the BBB endothelium under conditions mimicking physiological SS during the timeframe of subsequent experiments, in which mixed cultures are initiated 24 h after the induction of SS and analyses are performed thereafter starting at 1 h and running until 24 h (25 and 48 h of total time, respectively), immunofluorescence analysis of the TJ and AJ proteins, zonula occludens (ZO)-1 and β-catenin, respectively, was performed (Figure 1).

We observed that both junctional proteins are expressed by b.End5 cells, and that they are increasingly expressed at the membrane level along time (Figure 1A,B). Moreover, an increasing exposure to orbital rotation promoted a change in cell morphology, from rounded to elongated, with a progressive alignment of the cells. Semi-quantitative analysis revealed an increase in ZO-1 fluorescence intensity (*p* < 0.05, Figure 1C) and a reduction in the number of membrane gaps (*p* < 0.001, Figure 1D) at 48 h as compared with 25 h. An increase in β-catenin fluorescence intensity (*p* < 0.001, Figure 1E), together with an increase in cell elongation (*p* < 0.001; Figure 1F), was also noticeable after 48 h of orbital rotation in comparison with the 25 h patterns.

Altogether, these findings corroborate our previous results that orbital rotation is a mechanical stimulus that promotes the expression of junctional proteins, as well as their localisation at the plasma membrane, and improves BBB morphological features, in line with the SS effects in vivo.

### 2.2. Adherens and Tight Junctional Complexes Are Compromised during BMEC–BCC Interaction

**Figure 2:**
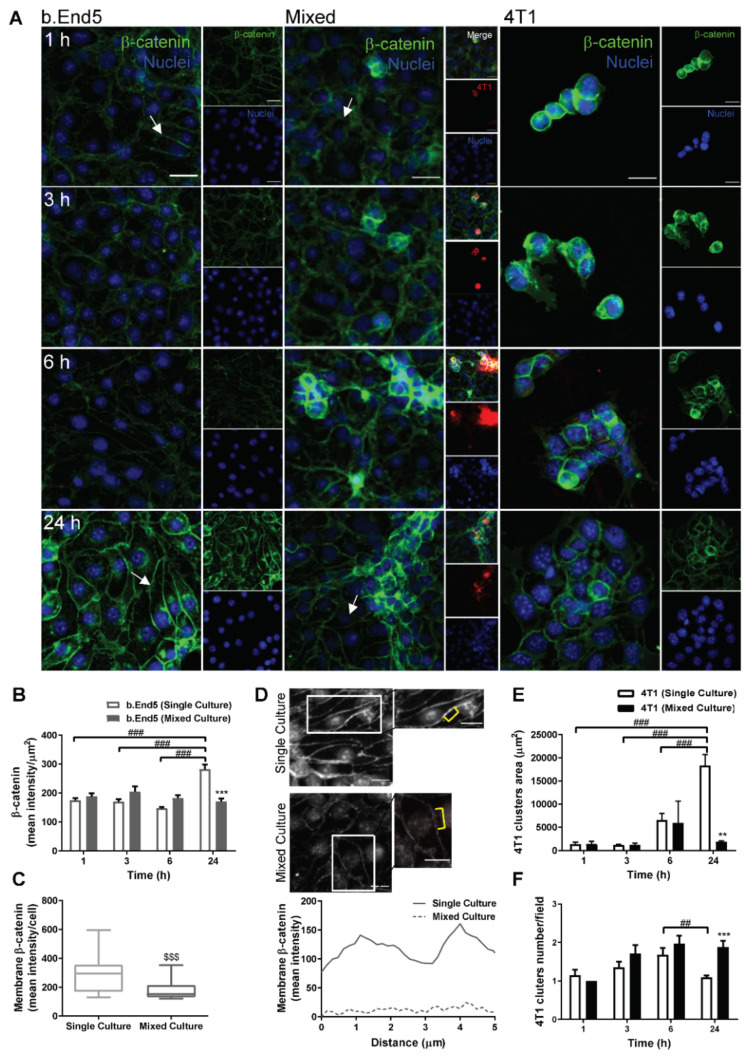
Adherens junctions are compromised during interaction between breast cancer cells and brain microvascular endothelial cells. Confluent monolayers of the brain endothelioma cell line b.End5 under physiological laminar non-pulsatile shear stress were exposed to 4T1 breast cancer cells (previously labelled with CellTracker™ DMTPX Red Dye) for 1, 3, 6 and 24 h and the expression of the adherens junction protein, β-catenin, in single and mixed cultures was evaluated by immunofluorescence analysis. (**A**) Analysis of the expression of β-catenin (green) revealed that the protein is present in both cell types, with a different cellular distribution in mixed cultures as compared with single ones, and further revealed a loss of elongation in b.End5 cells exposed to 4T1 cells (white arrows). Hoechst 33342 was used as counterstaining for nuclei (blue). Scale bar: 20 µm. Semi-quantitative analysis revealed (**B**) a decrease in endothelial β-catenin intensity in mixed culture at 24 h, (**C**) particularly notorious at the cell membrane, which was validated by the (**D**) plot profile analysis of the membrane region pointed out with the yellow brackets. Scale bar: 20 µm. The characterisation of 4T1 cells was performed by the quantification of the (**E**) area of tumoural clusters, which increased over time in single cultures, and (**F**) number of clusters, which decreased at 24 h in single culture. Data are given as means ± SEM (*n* = 3, 10 fields/condition). A one-way ANOVA was used to evaluate the significant differences within single and mixed cultures along time, represented by ^##^ *p* < 0.01 and ^###^ *p* < 0.001, and to evaluate the significant differences between single and mixed cultures at the same timepoint, represented by ** *p* < 0.01 and *** *p* < 0.001. A Mann–Whitney test was used to evaluate the significant differences between single and mixed cultures at 24 h of membrane β-catenin intensity, represented by ^$$$^ *p* < 0.001.

**Figure 3:**
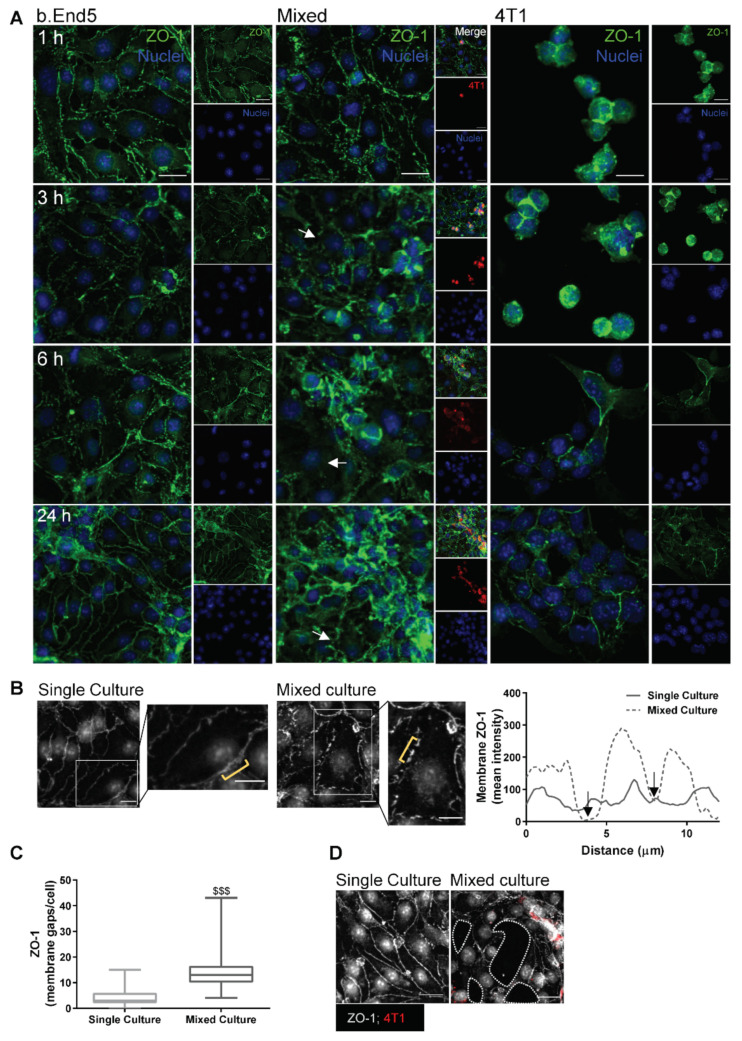
Tight junctions are compromised during interaction between breast cancer cells and brain microvascular endothelial cells. Confluent monolayers of the brain endothelioma cell line b.End5 under physiological laminar non-pulsatile shear stress were exposed to 4T1 breast cancer cells (previously labelled with CellTracker™ DMTPX Red Dye) for 1, 3, 6 and 24 h and the expression of the tight junction protein, zonula occludens-1 (ZO-1), in single and mixed cultures was evaluated by immunofluorescence analysis. (**A**) Analysis of the expression of ZO-1 (green) revealed that this protein is present in both cell types, with a different cellular distribution and disorganisation in b.End5 cells exposed to 4T1 cells (white arrows). Hoechst 33342 was used as counterstaining for nuclei (blue). Scale bar: 20 µm. (**B**) Analysis of membrane ZO-1 expression (grey) in b.End5 cells in mixed culture at 24 h revealed the presence of membrane gaps (insets and black arrows in the plot, which corresponds to the membrane region pointed out in yellow). Scale bar: 20 µm. (**C**) Semi-quantitative analysis revealed an increase in the number of membrane gaps in b.End5 cells in mixed cultures. (**D**) Inspection of the endothelial monolayer revealed holes (dotted lines) near 4T1 cells (red). Scale bar: 15 µm. Data are given as means ± SEM (*n* = 3, 10 fields/condition). Mann–Whitney test was used to evaluate the significant differences of membrane gaps, represented by ^$$$^ *p* < 0.001.

To establish the consequences of endothelial–malignant cells interaction on barrier integrity, we analysed the expression of β-catenin (Figure 2), an AJ protein fundamental to intercellular adhesion and barrier restricted permeability [16], as well as of ZO-1 (Figure 3), a TJ accessory protein essential to barrier properties’ maintenance [16,39].

The results showed that β-catenin is expressed by both b.End5 and 4T1 cells, in single and in mixed cultures (Figure 2A). They also revealed that BCCs led to a reorganization of the junctional protein expression at the membrane level in BMECs, as well as to an impairment of the elongated and aligned morphology, with b.End5 appearing to be more rounded (Figure 2A). Semi-quantitative analysis revealed that β-catenin expression in endothelial cultures increased at 24 h (*p* < 0.001 vs. 1, 3 and 6 h, Figure 2B), in line with the SS-induced effect on the expression of the junctional protein (Figure 1B), while in mixed cultures, such an increase was not observed along time. Importantly, after 24 h, β-catenin expression in b.End5 cells was significantly lower in mixed than in single cultures (*p* < 0.001, Figure 2B). Such a decrease was accompanied by a reduction in membrane β-catenin (*p* < 0.001, Figure 2C) and corroborated by the plot profile (Figure 2D), where almost no intensity is observed in the membrane of b.End5 cells in mixed cultures (dashed line) compared with single culture (solid line). Regarding 4T1 cells alone, they formed increasingly larger clusters (i.e., three or more 4T1 grouped cells) in single cultures along time (Figure 2E), whereas more clusters were observed in mixed cultures at 24 h (*p* < 0.01 vs. single culture, Figure 2F). Nevertheless, β-catenin, at both early (1 h) and later (24 h) timepoints, presented a similar distribution pattern in 4T1 cells in both single and mixed culture, localising mainly towards the cell membrane (Figure 2A).

Regarding TJ, we observed that both b.End5 and 4T1 express ZO-1 in single and mixed cultures (Figure 3). ZO-1 localised preferentially at the membrane level in single cultures of b.End5, whereas in mixed culture a progressive disorganisation of the ZO-1 labelling, with uneven and non-homogeneous staining at the cell membrane level and an unorganised endothelial monolayer, was observed (Figure 3A). An in-depth analysis of ZO-1 expression at 24 h revealed that this TJ is discontinuous along the cell membrane, corresponding to membrane gaps (Figure 3B). Accordingly, semi-quantitative analysis showed a significant increase in membrane gaps in b.End5 cells exposed to 4T1 cells (*p* < 0.001, Figure 3C). Importantly, after 24 h of contact with BCCs, the endothelium appeared disrupted, as confirmed by the presence of holes in the monolayer (Figure 3D). Regarding 4T1 cells, the distribution of ZO-1 presented a preferential localisation at the membrane level, though in mixed cultures it also appeared at the cytoplasm (Figure 3A).

Overall, these findings suggest that the interaction between the 4T1 and b.End5 cells compromises BBB endothelium integrity, affecting not only the AJs but also the TJs, pointing to a possible paracellular mechanism of tumour cells’ TEM.

### 2.3. BBB Transcellular Permeability Increases with BMEC–BCC Interaction

**Figure 4:**
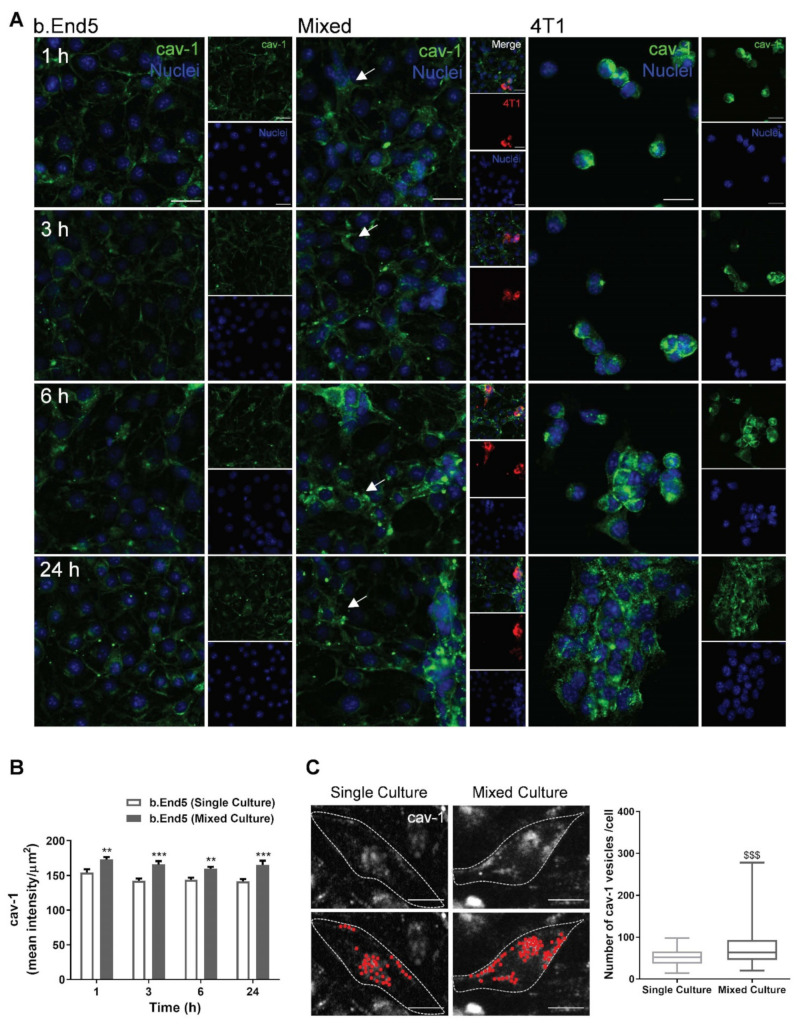
Vesicular trafficking alterations occur during breast cancer cell and brain microvascular endothelial cell interaction. Confluent monolayers of the brain endothelioma cell line b.End5 under physiological laminar non-pulsatile shear stress were exposed to 4T1 breast cancer cells (previously labelled with CellTracker™ DMTPX Red Dye) for 1, 3, 6 and 24 h and the expression of the main protein of caveolae, caveolin-1 (cav-1), in single and mixed cultures was evaluated by immunofluorescence analysis. (**A**) Analysis of the expression of cav-1 (green) revealed that the vesicular trafficking protein is present in both cell types, with a different cellular distribution and disorganisation in b.End5 cells (white arrows) exposed to 4T1 cells. Hoechst 33342 was used as counterstaining for nuclei (blue). Scale bar: 20 µm. (**B**) Semi-quantitative analysis revealed an increase in endothelial cav-1 intensity in mixed cultures vs. single culture. (**C**) Cav-1-positive vesicles were analysed in b.End5 cells in single and in mixed cultures at 24 h and representative images of the original and algorithm-based detected spots (red) are shown (up and down images, respectively); semi-quantitative analysis revealed an increase in the number of vesicles in mixed cultures as compared with single cultures. Scale bar: 10 µm. Data are given as means ± SEM (*n* = 3, 10 fields/condition). A one-way ANOVA was used to evaluate the significant differences between single and mixed cultures at the same timepoints, represented by ** *p* < 0.01 and *** *p* < 0.001. A Mann–Whitney test was used to evaluate the significant differences of the number of caveolae at 24 h represented by ^$$$^ *p* < 0.001.

Besides paracellular alterations observed regarding TJs and AJs, we further wanted to assess whether caveolae-mediated transcytosis is upregulated upon b.End5 exposure to 4T1 cells. To this end, the expression of the major protein constituent of caveolae, cav-1 [29], was examined (Figure 4).

We observed that cav-1 is expressed in both b.End5 and 4T1 cells (Figure 4A). Cav-1 in b.End5 cells was mainly observed at the membrane level in single culture, while in mixed cultures the expression of the vesicular protein in the cytosol was observed, in line with the occurrence of caveolae-mediated transcytosis. Semi-quantitative analysis revealed an increase in cav-1 in b.End5 cells in mixed cultures comparatively to single cultures in all timepoints (*p* < 0.01 at 1 and 6 h; *p* < 0.001 at 3 and 24 h, Figure 4B). Spot analysis of cav-1-positive vesicles at 24 h reinforced such observations, demonstrating an increase in the number of cav-1-positive vesicles in b.End5 cells when exposed to 4T1 cells (*p* < 0.001, Figure 4C).

These results point to the association between transcellular hyperpermeability (via an increase in cav-1 expression and caveolae number) and 4T1 cells’ transmigration across the BBB. Moreover, they reveal that cav-1 is expressed in “metastasis-like” clusters, in line with a pro-tumourigenic effect of the caveolae-associated protein.

### 2.4. The GJs Protein Cx43 Is Involved in BMECs-BCCs Interaction

**Figure 5:**
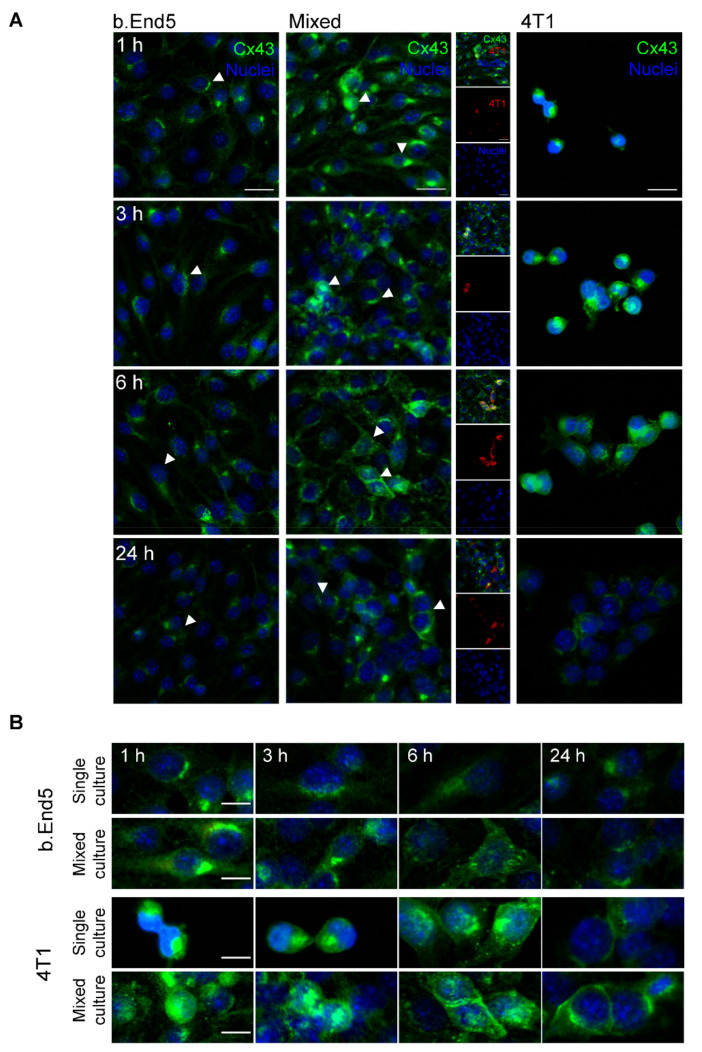
Alterations in the expression pattern of the gap junction protein connexin 43 (Cx43) occur during interaction between breast cancer cells and brain microvascular endothelial cells. Confluent monolayers of the brain endothelioma cell line b.End5 under physiological laminar non-pulsatile shear stress were exposed to 4T1 cells (previously labelled with CellTracker™ DMTPX Red Dye) for 1, 3, 6 and 24 h, and the expression of Cx43 in single and mixed cultures was evaluated by immunofluorescence analysis. (**A**) Analysis of the expression of Cx43 (green) revealed that this gap junction protein is present in both cell types. Hoechst 33342 was used as counterstaining for nuclei (blue). Scale bar: 20 µm. (**B**) One of each cell type is shown with a greater magnification (indicated by arrow heads in panel A) to better elucidate the differences observed in Cx43 expression and localisation in single and mixed cultures. Scale bar: 5 µm.

Considering that GJ intercellular communication has been considered a key mechanism in tumour cells’ migration and proliferation [36], the analysis of Cx43 during BCC and BMEC interaction was performed (Figure 5).

Our results revealed that both b.End5 and 4T1 in single and mixed cultures express Cx43, with specific patterns for each cell type in single and mixed cultures (Figure 5A). In endothelial cultures, Cx43 localised particularly in the perinuclear region, rather than at cell-to-cell contacts. After exposure to 4T1 cells, the perinuclear localisation was maintained but a redistribution throughout the cell was observed. Moreover, location of the protein in endothelial–tumour cell contact regions was detected. In single cultures of 4T1 cells, Cx43 was initially located in the perinuclear region (1 and 3 h), extending to the cytosol (6 h) and afterwards to cell-to-cell contacts (24 h), as clusters developed (Figure 5B). A similar profile is observed in 4T1 after contact with the endothelium, with the expression of Cx43 in tumour cell-to-cell contact later in time noticed (6 and 24 h, Figure 5B).

These data suggest that Cx43 is involved in the interaction between endothelial and tumour cells, as well as among tumour cells during the formation of tumour clusters.

### 2.5. BMEC–BCC Interaction Leads to Cytoskeleton Alterations

**Figure 6:**
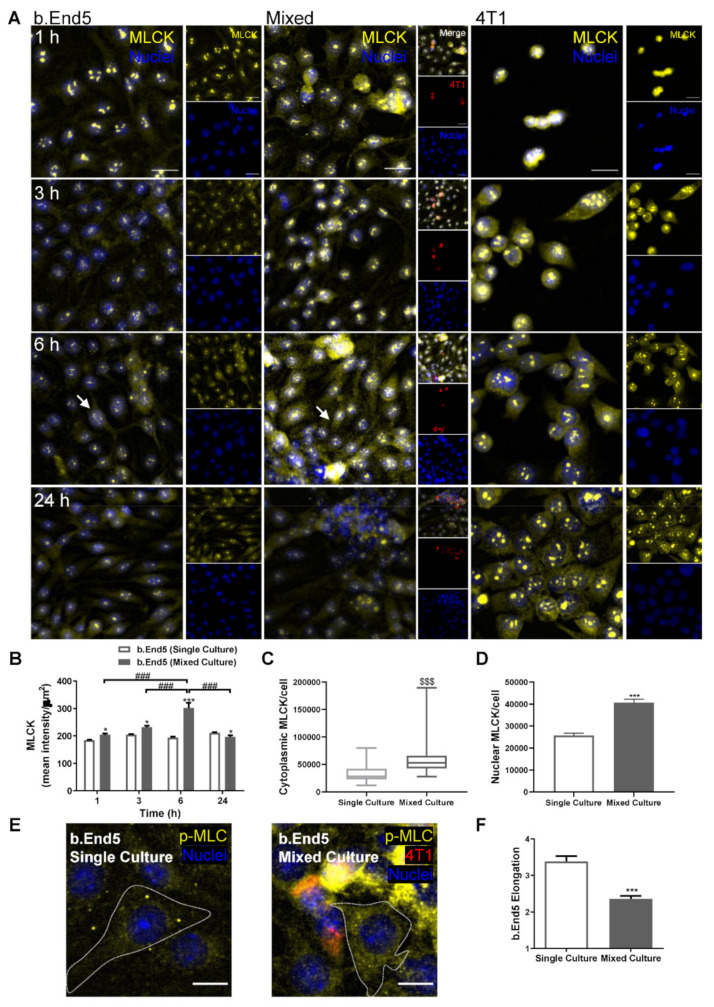
Interaction between breast cancer cells and brain microvascular endothelial cells leads to cytoskeleton rearrangement. Confluent monolayers of the brain endothelioma cell line b.End5 under physiological laminar non-pulsatile shear stress were exposed to 4T1 breast cancer cells (previously labelled with CellTracker™ DMTPX Red Dye) for 1, 3, 6 and 24 h and the expression of myosin light chain kinase (MLCK), as well as of phosphorylated myosin light chain (p-MLC), in single and mixed cultures was evaluated by immunofluorescence analysis. (**A**) Analysis of the expression of MLCK (yellow) revealed that the cytoskeleton-associated protein is present in both cell types, with a marked overexpression in b.End5 cells exposed to 4T1 cells at 6 h (as shown by the white arrows). Hoechst 33342 was used as counterstaining for nuclei (blue). Scale bar: 20 µm. (**B**) Semi-quantitative analysis revealed an increase in endothelial MLCK intensity in mixed cultures, particularly at 6 h. A significant increase in (**C**) cytoplasmic and (**D**) nuclear MLCK in mixed cultures compared with single ones was observed. (**E**) Cytoskeleton rearrangements were confirmed by the increase in p-MLC (yellow) observed in the cytoplasm of b.End5 cells (circumscribed by dotted line) exposed to 4T1 cells at 6 h. Hoechst 33342 was used as counterstaining for nuclei (blue). Scale bar: 10 µm. (**F**) Semi-quantitative analysis of the morphology of p-MLC stained b.End5 revealed a decrease in elongation upon incubation with 4T1 cells for 6 h. Data are given as means ± SEM (*n* = 3, 10 fields/condition). A one-way ANOVA was used to evaluate the significant differences within single and mixed cultures along time, represented by ### *p* < 0.001, and to evaluate the significant differences between single and mixed cultures at the same timepoints, represented by * *p* < 0.05 and *** *p* < 0.001. A Mann–Whitney test was employed to evaluate the significant differences in cytoplasmic intensity, represented by ^$$$^ *p* < 0.001. A two-tailed Student’s *t*-test was used to evaluate the significant differences in nuclear intensity and cell elongation, represented by *** *p* < 0.001.

To investigate the association of endothelial cytoskeleton rearrangements with BCCs’ transmigration, a cytoskeleton-associated protein, MLCK, reported to be involved in the transcellular intravasation of BCCs [28] and brain microvasculature hyperpermeability [40], was evaluated (Figure 6).

Both endothelial and tumour cells expressed MLCK, in single and mixed cultures, being evident at the cytoplasmatic level and in nuclear foci, as well as a marked overexpression in ECs in mixed cultures at 6 h of interaction (Figure 6A). Semi-quantitative MLCK fluorescence intensity analysis revealed that it was kept constant in b.End5 single cultures over time, whereas in mixed cultures an increase was observed, particularly notorious at 6 h (*p* < 0.001 vs. 1, 3 and 24 h), also being significantly higher compared to its expression in single culture at the same timepoint (*p* < 0.001, Figure 6B). Interestingly, the observed increase in the mean intensity appears to be mainly due to the cytoplasmatic MLCK overexpression (*p* < 0.001, Figure 6C), even though an increase in nuclei foci was also observed (*p* < 0.001, Figure 6D). Such an observation was corroborated by the increase in p-MLC cytoplasmatic intensity in mixed cultures (Figure 6E). Additionally, morphologic alterations were observed in b.End5 cells in mixed culture, with a decreased elongation of BMECs (*p* < 0.001; Figure 6F), supporting MLCK’s effect on cell contractility.

These results reinforce the occurrence of cytoskeleton alterations as a consequence of BCCs’ interaction with BMECs.

### 2.6. Adhesion-Related Signaling Pathway Activation Occurs during BMEC–BCC Interaction

**Figure 7:**
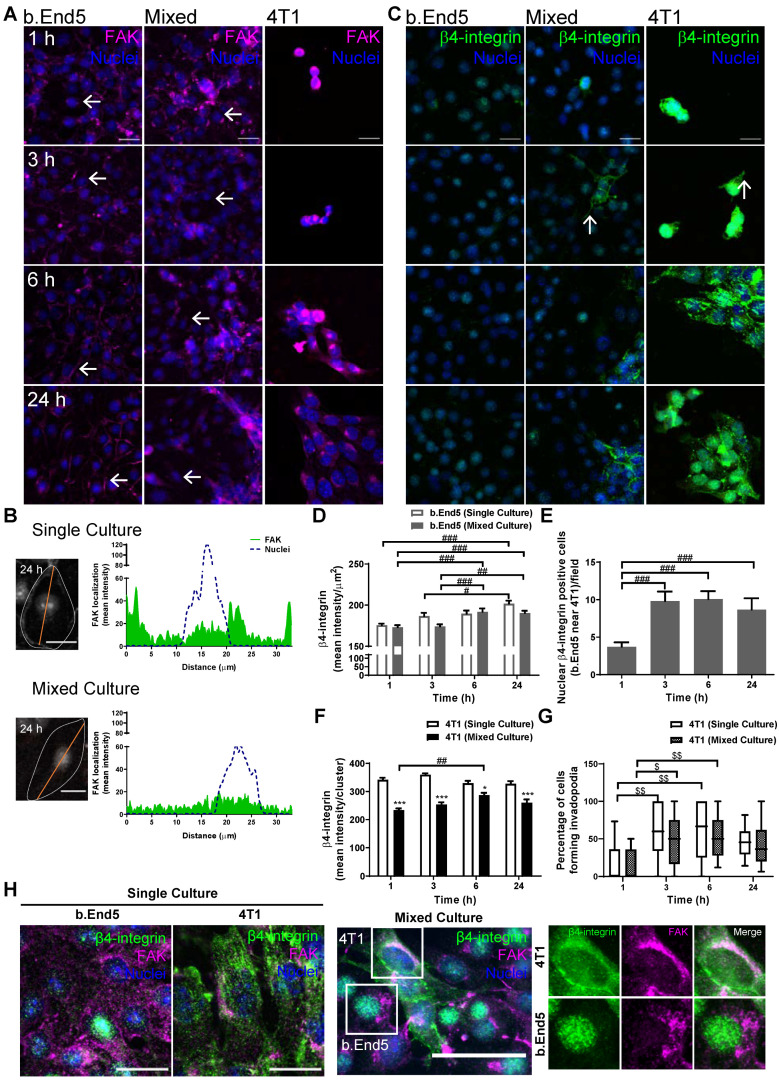
Interaction between breast cancer cells and brain microvascular endothelial cells leads to activation of adhesion-related signalling pathways. Confluent monolayers of the brain endothelioma cell line b.End5 under physiological laminar non-pulsatile shear stress were exposed to 4T1 breast cancer cells (previously labelled with CellTracker™ DMTPX Red Dye) for 1, 3, 6 and 24 h and the expression of focal adhesion kinase (FAK) and β4-integrin in single and mixed cultures was evaluated by immunofluorescence analysis. (**A**) Analysis of the expression of FAK (purple) revealed that the protein is present in both cell types, with an early overexpression, followed by a decrease in b.End5 cells exposed to 4T1 cells (white arrows). (**B**) Plot profiles showed notorious alterations in FAK (grey, cell border identified as dotted grey line) cell localisation and decreased expression in b.End5 in mixed culture as compared with single culture at 24 h. (**C**) Analysis of the expression of β4-integrin (green) showed that the protein is expressed in both cell types, with no changes in mixed cultures, and highlighted invadopodia formation in 4T1 cells in both single and mixed culture (white arrows). Hoechst 33342 was used as counterstaining for nuclei (blue). Scale bar: 20 µm. (**D**) Semi-quantitative analysis of β4-integrin expression revealed an increased content in b.End5 in single and mixed cultures along time. (**E**) Nuclear β4-integrin-positive b.End5 cells in close vicinity to 4T1 increased along time. (**F**) Semi-quantitative analysis of β4-integrin intensity per 4T1 clusters revealed a decrease in mixed cultures as compared with single ones. (**G**) Quantitative analysis of the number of 4T1 cells forming invadopodia revealed an increase post 3 h of culture in both single and mixed culture. (**H**) Double-labelling with β4-integrin and FAK depicted a notorious difference in the proteins’ cellular distribution in b.End5 and 4T1 cells both in single and mixed culture, shown by the colocalisation analysis revealing that both proteins colocalise in 4T1 cells (white coloration). Hoechst 33342 was used as counterstaining for nuclei (blue). Scale bar: 20 µm. Data are given as means ± SEM (*n* = 3, 10 fields/condition). One-way ANOVA was used to evaluate the significant differences within single and mixed cultures along time, represented by ^#^ *p* < 0.05, ^##^ *p* < 0.01 and ^###^ *p* < 0.001, and to evaluate the significant differences between single and mixed cultures at the same timepoints, represented by * *p* < 0.05 and *** *p* < 0.001. A Kruskal–Wallis test was used to evaluate the significant differences in the percentage of 4T1 cells forming invadopodia in single and mixed cultures along time, represented by ^$^ *p* < 0.05 and ^$$^ *p* < 0.01. A two-tailed Student’s *t*-test was used to evaluate the significant differences along time in b.End5 cells in mixed cultures with increased nuclear β4-integrin, represented by ^##^ *p* < 0.01 and ^###^ *p* < 0.001.

In order to disclose adhesion signalling involvement in BCCs’ interaction with the BBB endothelium, the expression of known adhesion-associated proteins, namely FAK [41] and β4-integrin [42], was evaluated (Figure 7).

Our results demonstrated that b.End5 and 4T1 cells expressed FAK and β4-integrin, both in single and mixed culture, presenting different expression and localisation patterns regarding cell type and culture conditions. In fact, FAK localisation changed from the membrane in b.End5 cultures towards the cytoplasm in b.End5 cells exposed to 4T1 cells. Moreover, a marked decrease in endothelial FAK expression was observed in 24 h mixed cultures, reflecting an impairment of the endothelial monolayer. The protein expression in 4T1 was mainly located in the perinuclear region, though some cytoplasm distribution was observed as well, both in single and in mixed cultures (Figure 7A). The subcellular localisation of FAK was corroborated by the plot profile analysis, which depicted a preferential location in the membrane and some perinuclear expression in b.End5 single cultures, which disappeared in mixed culture at 24 h (green filled line in the plots, Figure 7B).

As far as β4-integrin is concerned, its localisation in b.End5 cells appeared similar in both cultures, while in 4T1 cells its localisation changed along time, becoming more membrane/cytoplasmatic from 3 h onwards, particularly in mixed culture (Figure 7C). Additionally, the β4-integrin-positive membrane protrusions formed by 4T1 were noticeable as early as 3 h, in single and mixed culture (Figure 7C). Semi-quantitative analysis of β4-integrin labelling intensity (Figure 7D) revealed an increase along time in single culture (*p* < 0.001 at 24 h vs. 1 h; *p* < 0.05 at 24 h vs. 3 h), as well as in mixed culture (*p* < 0.001 at 6 h vs. 1 and 3 h; *p* < 0.001 at 24 h vs. 1 and 3 h), with no alterations in mixed versus single cultures, suggesting that the observed increase was a reflex of SS. It is important to mention that, in mixed cultures, β4-integrin nuclear expression in b.End5 cells in close proximity to 4T1 clusters appeared to be more intense (Figure 7C). This was corroborated by the semi-quantitative analysis of b.End5 cells with nuclear β4-integrin (Figure 7E), which highlighted an increase at 3 h (*p* < 0.001 vs. 1 h), sustained afterwards (*p* < 0.001 at 6 h vs. 1 h; *p* < 0.01 at 24 h vs. 1 h). Semi-quantitative analysis of β4-integrin in 4T1 cells revealed a general decrease in the mean intensity per cluster in mixed as compared with single cultures (*p* < 0.001, Figure 7F). Quantitative analysis of the number of 4T1 cells forming invadopodia (Figure 7G) revealed an increase from 3 h of single culture (*p* < 0.01 at 1 h vs. 3 and 6 h) as well as in mixed culture (*p* < 0.05 at 3 h vs. 1 h; *p* < 0.01 at 6 h vs. 1 h).

It is known that, in tumour cells, FAK and β4-integrin interact, prompting cell migration and invasiveness [13]. Thus, we performed a double-labelling of FAK and β4-integrin. As shown in Figure 7H, FAK and β4-integrin are co-expressed in the perinuclear region of 4T1 cells in single and particularly in mixed cultures, in contrast with b.End5 cells that exhibit a distinct location of each protein, with the former in the perinuclear region and the latter in the nuclei. These observations attest the proximity of both proteins in malignant cells, in line with the important role of their interaction in tumourigenesis.

Our data suggest that both FAK and β4-integrin are involved in the processes of 4T1 interaction with the BBB endothelium.

## 3. Discussion

**Figure 8:**
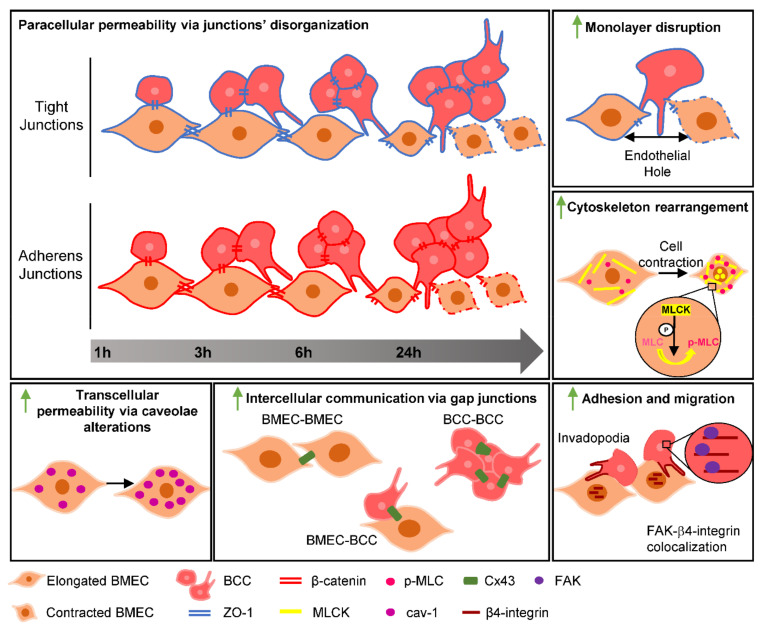
Schematic representation of the players involved in the interaction between brain microvascular endothelial cells (BMECs) and breast cancer cells (BCCs). Along time, BCCs form clusters that increase in size. BMECs, upon contact with BCCs, suffer several alterations, where junctional impairment, indicated by β-catenin and zonula occludens (ZO)-1, and endothelial monolayer hole formation are noticeable at later timepoints. Additionally, cytoskeleton rearrangements occur through an increase in myosin light chain kinase (MLCK) and phosphorylated myosin light chain (p-MLC), resulting in BMEC contraction and transcytosis upregulation shown by the increase in the vesicular content of caveolin-1 (cav-1), supporting endothelial paracellular and transcellular hyperpermeability involvement in BCCs’ transmigration. BCCs present migratory properties seen by the formation of invadopodia. The expression alterations in connexin 43 (Cx43) suggest that this gap junction protein is involved in the interaction between BMECs and BCCs. β4-Integrin in invadopodium and β4-integrin-focal adhesion kinase (FAK) colocalisation point to their role in intercellular adhesion and BCC migration.

Despite the fact that BCCs’ intravasation mechanisms are well established [43], BCCs extravasation is a process that is not yet fully comprehended, in particular regarding TEM and the players involved. Tumour cells’ TEM across the BBB has been described to occur either by the paracellular route [22] or by the transcellular one [23], but the exact mechanisms and intervenients in BCCs’ extravasation are not yet fully disclosed. A recent study by our team provided comprehensive evidence regarding in vivo BC brain metastasis formation hallmarks [19]. In the present work, we further disclosed players and mechanisms involved, highlighting the role of AJs, TJs, GJs, caveolae, the cytoskeleton, kinases, and adhesion proteins along in vitro BCCs’ interaction with BMECs, pointing to their hub role along extravasation (schematically depicted in Figure 8), mirroring most of the in vivo observations [19].

A novel cellular system replicating BCCs’ interaction with BBB endothelial cells during the process of the extravasation of malignant cells into the brain was developed. This in vitro model encompasses b.End5 monolayers, mimicking the BBB, cultured under orbital rotation to recapitulate physiological SS in mixed cultures with highly metastatic triple-negative BCCs, 4T1 cells, fluorescently labelled to distinguish individual cell types. As a result of the mechanical stimulus, BMEC cultures present an in vivo-like morphology, becoming more elongated and aligned, increasing the expression of junctional proteins, in line with previous observations by our group [17]. In this system, 4T1 cells appear to behave differently comparatively with single culture, where “metastasis-like” clusters are formed on top of the endothelial monolayer, which increase in size along time. Although it is known that the major cause of tumour cell loss during the metastatic process is the circulation in the bloodstream [44], mimicked by SS in our system, cluster formation seems to be an adaptive response of 4T1 to better survive, adhere and invade the endothelium. In fact, cluster formation was already described to occur in vivo, as a defence mechanism of BCCs, facilitating the metastatic process [45]. Contrarily to single migration, the association of cluster formation with the collective migration of tumour cells that seems to be a way of saving energy. In these clusters, the so called “leader cells” are generally located at the periphery, being responsible for the guidance of cluster migration and tending to be more aggressive [46,47]. Moreover, this type of migration seems to rely on the formation of membrane protrusions in the leader cells at the cluster’s front [48]. Indeed, we observed that cells at the cluster’s periphery are the ones that present some footlike projections, such as invadopodia, known to be associated with tumour cell migration, suggesting that collective migration is a mechanism acquired by 4T1 cells to be able to migrate and invade the BBB. Although collective migration has been described for the intravasation of BCCs [49], as well as in studies of BCCs’ extravasation through ECs in microfluidic devices [50], the present results suggest that collective migration may also be associated with TEM of BCCs across BMECs. This suggestion is supported by a previous work showing that cell–cell junctions are important for aggregate/cluster cohesion [51], and the present results showing that 4T1 cells express the junctional proteins, β-catenin and ZO-1.

It is known that both TJ and AJ proteins play a crucial role in BBB tightness and selective permeability maintenance [16]. However, the disruption of junctional complexes in the context of brain metastasis formation has been reported [52,53], leading to an increase in brain permeation, a phenomenon that has also been associated with BCCs’ paracellular transmigration [19]. Indeed, a decrease in the TJ and AJ proteins, claudin-5 and β-catenin, respectively, was observed along the formation of 4T1-derived metastasis in vivo, with a concomitant increase in the BBB permeability shown by thrombin infiltration into the brain parenchyma, particularly in advanced stages of metastases development [19]. In line with these features observed in a mouse model, the present in vitro studies revealed that b.End5 cells present a decrease in β-catenin and ZO-1 expression at the plasma membrane, where the ZO-1 staining disclosed the presence of membrane gaps and the presence of holes in the endothelial monolayer. Interestingly, endothelium disruption depends on both endothelial and tumour cells. In the case of interaction between mice endothelioma cell line, b.End3, and the BCC line, MDA-MB-231, for 4-8 h, both adherent and transmigrated BCCs were observed, with endothelial TJ impairment in contact regions due to the partial and total disruption of membrane ZO-1, respectively [54]. On the other hand, in melanoma cells, a disruption of EC–EC junctions is observed early in time post-endothelial–tumour cell contact with the increase in holes in the endothelium near the tumour cells [55]. Similarly, in our study, endothelial disruption in the vicinity of BCCs occurs, although later in time compared with both studies. Due to the alterations observed at the endothelial level, we can point to a likely paracellular mechanism of BCCs’ transposition of the BBB.

Although the present results indicate that the paracellular route is affected by b.End5–4T1 interaction, the transcellular pathway cannot be discarded. This pathway has been recognised for leucocytes’ TEM, namely across the BBB, with the involvement of cytoskeleton remodelling, pore formation within ECs, and protrusion extension of leukocytes [56], as well as in BC brain metastasis formation [23]. Among the proteins described to have a role in the transcellular pathway and endothelial permeability, cav-1 is a key player [30,57]. Cav-1 is the main protein constituent of caveolae [16], which are believed to function not only as vesicular transporters, but also as a signal platform that regulates cell proliferation, differentiation, and metastases development [58,59]. The increase in cav-1 has been associated with endothelial hyperpermeability, particularly at the BBB, being an early event compared with TJ impairment [57,60]. Our results showed an increase in cav-1 expression in BMECs in mixed cultures compared with single ones, as well as a significant increase in the caveolae number later in time, coincident with junctional disruption. Consistently, cav-1 upregulation was also observed in in vivo BC brain metastasis formation, particularly in blood vessels in the vicinity of BCCs and of metastatic brain lesions [19]. The parallelism between the findings attained in the present study and the ones obtained in vivo reinforces the physiological relevance of our in vitro model for mirroring the in vivo disease pathobiology.

Endothelial cytoskeleton rearrangements have been correlated with cancer cell transmigration, being particularly described during intravasation through vascular ECs, where an endothelial actomyosin circumferential pore is formed, regulated by MLCK phosphorylation of MLC and prompting cytoskeleton rearrangements and actin fibre formation [28,61]. For extravasation, the preference of BCCs for transcellular migration across BMECs has recently been shown, occurring by diapedesis through ECs’ body [23]. Although the authors do not explore the mechanism, they propose the further study of cytoskeleton rearrangement involvement [23]. In conformity, our results demonstrate an increase in endothelial MLCK after 6 h of exposure to BCCs, with a concomitant increase in p-MLC, implying that cytoskeleton rearrangements are occurring in BMECs in response to BCCs, with possible association with transcellular transmigration. Interestingly, a decrease in the TJ protein ZO-1 following actin remodelling through MLC phosphorylation 4–8 h post BMEC–tumour cell contact, associated with junctional opening, was reported [53]. These observations corroborate our own of a ZO-1 decrease after MLCK overexpression and MLC phosphorylation in BMECs, suggesting that cytoskeleton rearrangements are associated with endothelial junctional opening during BCCs’ TEM. Additionally, we highlight the observation of MLCK nuclear foci, particularly in BMECs, which decreased in contact with BCCs. Nuclear MLCK was related to the dynamics of the nuclear cytoskeleton and the regulation of gene transcription in human colonic smooth muscle cells [62], whereas nuclear myosin seems to be involved in nuclear actin shifting and compaction associated with the migration and TEM of BCCs during intravasation [63]. Our present results are in line with those conclusions, once alterations in endothelial MLCK and p-MLC upon contact with BCCs point to cytoskeleton alterations, which enhance BCC transmigration associated with an increase in BMEC permeability. Altogether, our results support the involvement of MLCK and cav-1 in the endothelium hyperpermeability and BCCs’ transcellular migration.

The role of GJs in intercellular communication in numerous physiological cellular functions, especially in proliferation control, accounts for the growing attention in the field of oncology [34], either between tumour cells in a metastatic environment, or even the crosstalk that can be established between BMECs and BCCs [64]. The relevance of Cx43 for the extravasation process of BCCs was previously demonstrated both in pulmonary [65] and in brain endothelium, where the inhibition of this GJ protein was associated with metastases formation inhibition [64]. Consistent with these findings, overexpressed Cx43 in BCCs promoted cell migration [66]. Here, we demonstrated that Cx43 is expressed by BMECs and BCCs and undergoes a subcellular redistribution upon interaction, pointing to a possible role of this protein in the interaction between the two cell populations. An increase in Cx43 expression was observed in BCC contact regions, particularly in well-established 4T1 clusters at later timepoints, pointing to the importance of inter-BCC communication for cluster formation and maintenance. Such intercellular communication was observed by us in an in vivo model of BC brain metastases [19], where Cx43 was shown to localise at both BCC–BCC and BCC–BMEC contact points. These observations point to a role of Cx43 for TEM across the BBB.

Integrins and adaptor molecules such as FAK, besides their adhesion and structural function, are also involved in signalling pathways, regulating several cellular mechanisms such as proliferation and migration [67]. FAK is a non-receptor tyrosine kinase and the most relevant signalling molecule in focal adhesion assembly and disassembly [68]. Moreover, it was described to have a pleiotropic action in tumour cell survival, proliferation, invasion, and metastasis formation through activation by integrin signalling [11,68], while in ECs it plays a key role in adhesion, angiogenesis and vascular permeability [69,70]. Interestingly, FAK has been associated with endothelial permeability regulation in pulmonary ECs, as endothelial FAK loss upon thrombin stimulus promoted a sustained decrease in transendothelial electric resistance [71]. Moreover, the activation of FAK and proto-oncogene tyrosine-protein kinase Src in ECs was shown to promote endothelial permeability through junctional impairment [70], particularly in response to tumour cell contact [15]. The herewith found overall decrease in FAK in prolonged BMEC exposure to BCCs, taken together with the junctional impairment observed in BMECs, suggests that FAK is associated with tumour transmigration by increasing endothelial permeability.

In epithelial cells, α6β4-integrin signalling is known to relate to its dissociation from the basement membrane and translocation to the cytoplasm and nucleus, promoting the activation of pathways, such as phosphoinositide 3-kinase and extracellular signal-regulated kinase [72]. Similarly, in cancer and particularly in BC, upon α6β4-integrin phosphorylation (at β4 subunit level in the cytoplasmatic tail), integrin release from the hemidesmosome occurs, perpetuating invasive signalling and promoting migration, invasion, proliferation, and tumourigenesis [73,74]. Curiously, in vascular ECs, the translocation of β4-integrin into the nucleus has been associated with cell apoptosis [75]. The present study reports an alteration of β4-integrin to nuclear sub-localisation in b.End5 cells in the proximity of 4T1 cells, which is consistent with either the loss of cellular adhesion and migration activation [72] or with the activation of cell death processes [75] that could account for the endothelial holes observed at latter stages of BMEC–BCC contact. Additionally, we showed an increase in β4-integrin in 4T1 cells, until 6 h of contact with the endothelium, which suggests a potential involvement of this protein in the migration and invasive mechanisms. In fact, it has been demonstrated that cancer cells can extend invadopodia when transmigrating through vessels [20], where some of the involved proteins described include integrins and FAK [76,77]. Furthermore, the preference for invadopodia to initiate where both FAK and integrins associate has been reported, particularly the α5β1-integrin, leading to signalling activation during invadopodium initiation and maturation [76]. Studies have shown that integrins participate in invadosome formation and progression; however, only β1 and β3 have been described [76]. Here, we suggest that β4-integrin is involved in invadopodia establishment and ultimately enhances the invasiveness phenotype of BCCs. Interestingly, we observed such a phenotype as early as at 3 h of BCC–BMEC contact. Such invadopodium extension is particularly evident through β4-integrin staining, leaving the question of whether this protein could have a role in this process. Importantly, authors have already described the recruitment of FAK by β4-integrin, and the consequent formation of the complex FAK/β4-integrin, with FAK activation, driving protein kinase B (Akt) signalling regulation in cancer [13,78]. We have also recently shown that FAK/β4-integrin colocalisation appears to be pivotal for in vivo BC brain metastasis establishment [19]. In accordance, here we found that, together with FAK, BCCs also express β4-integrin, which co-expressed at advanced stages of the extravasation process, suggesting the activation of these signalling pathways during metastasis formation.

## 4. Materials and Methods

### 4.1. Cell Culture Conditions

Mouse BALB/c brain endothelioma cell line b.End5 (ECACC, Salisbury, UK) was used as a simplified BBB in vitro model. b.End5 cells were grown in Dulbecco’s modified Eagle’s medium (DMEM, Gibco, Life Technologies, New York, NY, USA) supplemented with 10% (*v/v*) foetal bovine serum (FBS, Biochrom AG, Berlin, DE), 1% (*v/v*) non-essential amino acids (Biochrom AG, Berlin, DE), 2 mM L-glutamine (Biochrom AG, Berlin, DE), 1 mM sodium pyruvate (Biochrom AG) and 1% (*v/v*) antibiotic–antimycotic solution (Sigma Aldrich, St. Louis, MO, USA).

The murine mammary carcinoma triple-negative 4T1 cell line (ATCC, Middlesex, UK) was also used. 4T1 cells were cultured in RPMI 1640 (Sigma Aldrich, St. Louis, MO, USA) supplemented with 2 mM L-glutamine and 5% (*v/v*) FBS. Both cell lines were maintained at 37 °C in humid atmosphere enriched with 5% CO_2_.

### 4.2. SS Application

Confluent monolayers of b.End5 cells were exposed to laminar non-pulsatile SS, achieved by orbital rotation, as previously described [17], using an orbital shaker (Grant Bio Orbital shaker PSU-10i, Grant Instruments, Cambridge Ltd., Royston, UK) positioned inside the incubator. The SS applied was estimated using the following equation:

$$\tau_{w}=\alpha \sqrt{\rho \eta {\left(2\pi f\right)}^{3}},$$

where τw is the SS value (dyn/cm^2^), α is the orbital radius of rotation of the shaker (0.5 cm), ρ is the density of the cell culture medium (1.01 g/cm^3^), η is the cell medium viscosity (0.0075 dyn/cm^2^ at 37 ℃), and f corresponds to rotations per second (rps) [79,80]. Physiological magnitudes of SS such as 1.5 dyn/cm^2^ have been reported [81,82] and were used in our assays, corresponding to a rotational frequency of 1.67 rps (100 rpm). A progressive ramping was made with a 0.17 rps (10 rpm) increase every 30 min until reaching physiological SS. SS conditions were maintained for 48 h.

### 4.3. BC Brain Metastasis Formation In Vitro Model Establishment

**Figure 9:**
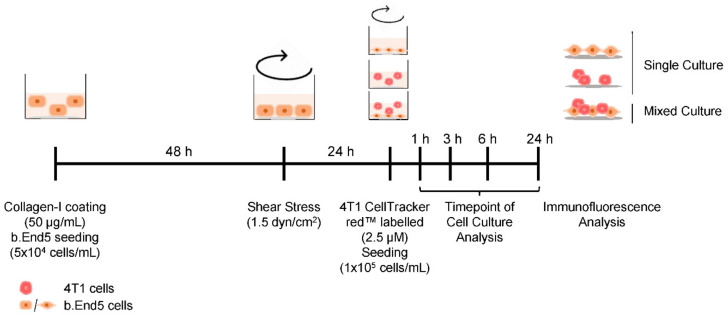
Schematic representation of the experimental design of the mouse breast cancer brain METABLE 5. was seeded at the concentration of 5 × 10^4^ cells/mL onto collagen-I coated coverslips (50 μg/mL) to allow a confluent monolayer formation during 48 h, after which physiological laminar shear stress (1.5 dyn/cm^2^) was applied by orbital rotation. After 24 h, mixed culture was initiated by the seeding of 1 × 10^5^ cell/mL murine mammary carcinoma triple-negative cells (4T1) previously labelled with CellTracker™ DMTPX Red Dye (2.5 μM) onto b.End5 monolayers. Single cultures (b.End5 and 4T1 cells) were run in parallel, as controls. Cell cultures were fixed at 1, 3, 6 and 24 h for immunofluorescence analysis.

As an in vitro model that mimics the BC brain metastasis development, mixed cultures of b.End5 and 4T1 cells were implemented. b.End5 cells (5 × 10^4^ cells/mL) were plated onto glass coverslips covered with rat tail collagen I (Corning, New York, NY, USA) at 50 µg/mL. After 48 h, physiological SS was applied for 24 h. In order to distinguish both cell populations, 4T1 cells were labelled with CellTracker™ Red CMTPX Dye (2.5 µM; Thermo Fisher Scientific, Waltham, MA, USA), in DMEM, and then plated (1 × 10^5^ cells/mL) on top of b.End5 monolayers. Mixed cultures were kept on SS conditions for 1, 3, 6 and 24 h, timepoints after which cells were fixed with 4% (*w*/*v*) paraformaldehyde (PFA, Sigma-Aldrich, St. Louis, MO, USA) in phosphate-buffered saline (PBS) for 20 min at room temperature. Assays were run in parallel for each cell type alone, as controls. The experimental design is depicted in Figure 9.

### 4.4. Immunofluorescence

Phenotypic alterations in endothelial and tumour cells in single and mixed cultures along time were evaluated by immunofluorescence analysis of junctional proteins (ZO-1, β-catenin, Cx43), transcellular transport-associated protein (cav-1), cytoskeleton-associated proteins (MLCK and p-MLC) and adhesion-associated proteins (FAK and β4-integrin). Following fixation, cells were permeabilised for 5 min, blocked for 60 min at room temperature, and incubated overnight at 4 °C with the primary antibodies and thereafter with the corresponding secondary antibodies for 60 min at room temperature, in the dark, as specified in Table 1. Both primary and secondary antibodies were diluted in corresponding blocking solutions. Nuclei were counterstained with Hoechst 33342 dye (Thermo Fisher Scientific; 1:1000 in PBS) for 10 min at room temperature. Cells were washed three times with PBS between incubations. Methanol dehydrated cells were then mounted in microscopy slides with DPX (Merck Millipore, Burlington, MA, EUA), properly dried and stored at 4 °C until image acquisition.

### 4.5. Image Acquisition and Data Analysis

Immunolabellings were examined using an Olympus BX60 microscope equipped with Olympus U-RFL-T Mercury lamp and Hamamatsu Orca R2 cooled monochromatic CCD camera, using 40x and 100x oil objectives.

For all stainings, 10 fields per condition were analysed. Data analysis was performed using ImageJ 1.29x software (National Institutes of Health, Bethesda, MD, USA), namely for the definition of quantification area, evaluation of mean intensity and their representation by plot profile, cell cluster number and area, as well the number of invadopodia forming cells. Icy (Institute Pasteur and France BioImaging, Paris, France) software was employed for the quantification of membrane and nuclear mean intensity, cytoplasmatic and total cell intensity, evaluation of cell morphology parameters such as cell elongation, and quantification of gaps in membrane staining (detailed in Table S1).

### 4.6. Statistical Analysis

Results were analysed using GraphPad Prism^®^ 6.0 (GraphPad Software, San Diego, CA, USA) and are expressed as mean ± SEM. The results represent the average of three independent experiments (*n* = 3). Two-tailed Student’s *t*-test and one-way ANOVA (parametric test for data with normal distribution) or a Mann–Whitney test or a Kruskal–Wallis test (non-parametric tests for data with an abnormal distribution, α = 0.05) was performed for comparisons between conditions and timepoints (Table S1). Statistically significant differences were considered when *p* < 0.05.

## 5. Conclusions

In this work, we implemented a new in vitro model to study BCCs’ interaction with the BBB endothelium, encompassing the effect of physiological SS, which represents an invaluable tool for studies of malignant cells’ extravasation across brain microvasculature and brain metastases formation. The results obtained allowed a comprehensive understanding of the players and signalling molecules involved in BCC–BMEC interaction, differentiating the molecular alterations occurring in malignant cells from those in BBB ECs. Importantly, it provided novel insights into conflicting or so far undetermined aspects of the extravasation process. We can conclude that exposure to BCCs leads to increased paracellular and transcellular permeability of the BBB ECs, culminating in severe disruption of the monolayer integrity, events associated with cytoskeleton alterations. On the other hand, invadopodia formation in BCCs via FAK and β4-integrin highlights its pro-metastatic role, where adhesion molecules appear as key determinants of the extravasation process. Overall, the findings reported here disclose possible targets for modulation in order to devise strategies to prevent the extravasation of BCCs into the brain and, thus, to avoid the formation of uncurable metastases.

## Figures and Tables

**Table 1:** Summary of the experimental conditions for immunofluorescence analysis.

Target Protein	Permeabilisation	Blocking	Primary Antibody	Secondary Antibody
**β-catenin**	0.3% Triton X-100	3% BSA	β-catenin (1:100) Thermo Fisher Scientific, #71-2700, Rabbit	Alexa Fluor^®^ 488 (1:500) Thermo Fisher Scientific, #A21206, Goat Anti-Rabbit
**β4-Integrin**	0.3% Triton X-100	3% BSA	β4-integrin (1:50) Santa Cruz Biotechnology, #sc-514426, Mouse	Alexa Fluor^®^ 488 (1:500) Thermo Fisher Scientific, #A11001 Goat Anti-Mouse
**Cav-1**	0.1% Saponin in 3% BSA	0.1% Saponin in 3% BSA	Caveolin-1 (1:100) Cell Signaling, #3238S, Rabbit	Alexa Fluor^®^ 488 (1:500) Thermo Fisher Scientific, #A21206, Goat Anti-Rabbit
**Cx43**	0.3% Triton X-100	3% BSA	Cx43 (1:50) Thermo Fisher Scientific, #35-5000, Mouse	Alexa Fluor^®^ 488 (1:500) Thermo Fisher Scientific, #A11001 Goat Anti-Mouse
**FAK**	0.3% Triton X-100	3% BSA	FAK (1:200) Abcam, #ab131435, Rabbit	Alexa Fluor^®^ 488 (1:500) Thermo Fisher Scientific, #A21206, Goat Anti-Rabbit
**MLCK**	0.3% Triton X-100	3% BSA	MLCK (1:100) Thermo Fisher Scientific, #PA515177, Rabbit	Alexa Fluor^®^ 488 (1:500) Thermo Fisher Scientific, #A21206, Goat Anti-Rabbit
**p-MLC**	0.3% Triton X-100	3% BSA	p-MLC (1:400) Thermo Fisher Scientific, #MA5-15163, Mouse	Alexa Fluor^®^ 488 (1:500) Thermo Fisher Scientific, #A11001 Goat Anti-Mouse
**ZO-1**	0.3% Triton X-100	3% BSA	ZO-1 (1:200) Thermo Fisher Scientific, #40-2200, Rabbit	Alexa Fluor^®^ 488 (1:500) Thermo Fisher Scientific, #A21206, Goat Anti-Rabbit

BSA, bovine serum albumin; cav-1, caveolin-1; cx43, connexin 43; FAK, focal adhesion kinase; MLCK, myosin light chain kinase; p-MLC, phosphorylated myosin light chain; ZO-1, zonula occludens-1.

## Data Availability

Not applicable.

## Supplementary Materials

The following are available online at https://www.mdpi.com/article/10.3390/ijms22137057/s1, Table S1: Data analysis of all studied parameters.

## Abbreviations

AJ	Adherens Junction
BBB	Blood–Brain Barrier
BC	Breast Cancer
BCCs	Breast Cancer Cells
BMECs	Brain Microvascular Endothelial Cells
BSA	Bovine Serum Albumin
Cav-1	Caveolin-1
Cx43	Connexin 43
ECs	Endothelial Cells
FAK	Focal Adhesion Kinase
FBS	Foetal Bovine Serum
GJ	Gap Junction
MLC	Myosin Light Chain
MLCK	Myosin Light Chain Kinase
PBS	Phosphate Buffer Solution
p-MLC	Phosphorylated Myosin Light Chain
SS	Shear Stress
TJ	Tight Junction
TEM	Transendothelial Migration
ZO-1	Zonula Occludens-1
