# Supplementary material for: Behind Brain Metastases Formation: Cellular and Molecular Alterations and Blood–Brain Barrier Disruption
Source: Int J Mol Sci. 2021 Jun 30;22(13):7057. doi: 10.3390/ijms22137057 (PMC8268492; doi:10.3390/ijms22137057)
Supplement: Supplementary file 1 [file ijms-22-07057-s001.zip › ijms-1251379-supplementary.pdf]

Table S1 | Detailed data analysis of all studied parameters.

Figure	Marker	Cell type	Marker	Software used	Analysis	Statistics
Figure 1	β-catenin	**b.End5**	Mean Intensity	ImageJ	Measured the mean intensity in 500x500 pixel square.	Two-tailed student's *t* -test
			Elongation	Icy	5 cells per field were circumscribed to obtain the elongation value:1 stand for a perfect circle. The higher the value, the more elongated is the cell.	Mann Whitney test
	ZO-1	**b.End5**	Mean Intensity	ImageJ	Measured the mean intensity in 500x500 pixel square.	Two-tailed student's *t* -test
			Membrane Gaps	Icy	5 cells were selected per field. The membrane gaps on ZO-1 staining were manually counted.	Mann Whitney test
Figure 2	β-catenin	**b.End5**	Mean Intensity	ImageJ	Measured the mean intensity in 500x500 pixel square.	One-way ANOVA
			Membrane Intensity	Icy	5 cells per field were circumscribed and the membrane mean intensity is measured.	Mann Whitney test
			Plot Profile	ImageJ	In a representative cell, a line of 5 μm was draw on top of the membrane staining and a profile of intensity was obtained in the respective interest protein channel to elucidate the intensity or presence of the protein in the membrane region.	
		**4T1**	Cluster Number	Image J	Number of clusters (≥ **3 cells**) was quantified manually.	One-way ANOVA
			Cluster Area		Measured the area of circumscribed clusters **(**≥ **3 cells**).	One-way ANOVA
Figure 3	ZO-1	**b.End5**	Plot Profile	ImageJ	In a representative cell, a line of 13 μm was draw on top of the membrane staining and a profile of intensity was obtained in the respective interest protein channel to elucidate the intensity or presence of the protein in the membrane region.	~~One-way ANOVA~~
			Membrane Gaps	Icy	5 cells were selected per field.<br>The membrane gaps on ZO-1 staining were manually counted.	Mann Whitney test
Figure 4	Cav-1	**b.End5**	Mean Intensity	ImageJ	Measured the mean intensity in 500x500 pixel square.	One-way ANOVA
				Icy	Spot detector in 5 representative cells per field was performed to identify the number of caveolae.	Mann Whitney test
Figure 6	MLCK	**b.End5**	Mean Intensity	ImageJ	Measured the mean intensity in 500x500 pixel square.	One-way ANOVA
			Cytoplasmic Total Intensity (at 6 h)	Icy	5 cells per field were circumscribed and the mean intensity of the entire cell was measured, as well as the cell nuclei. The values of mean intensity were multiplied by its respective area, to obtain the total intensity of cells	Mann Whitney test

					and the respective nuclei, and the cytoplasmic content was quantified by the difference between total cell intensity and total nuclear intensity.	
			Nuclear Total Intensity (at 6 h)		5 cells per field were circumscribed and the mean intensity of the entire cell was measured, as well as the cell nuclei. The values of mean intensity were multiplied by its respective area, to obtain the total intensity of the nuclei.	Two-tailed student´s *t*-test
	p-MLC	**b.End5**	Cell elongation	Icy	5 cells per field were circumscribed to obtain the elongation value:1 stand for a perfect circle. The higher the value, the more elongated is the cell.	Two-tailed student´s *t*-test
Figure 7	FAK	**b.End5**	Plot Profile		In a representative cell, a line of 33 μm was drawn through the cell and a profile of intensity was obtained for both nuclei and protein channel, in order to be able to identify the localization of the protein along the cell.	One-way ANOVA
	β4-integrin		Mean Intensity	ImageJ	Measured the mean intensity in 500x500 pixel square.	One-way ANOVA
			Number of cells with Nuclear Staining		Number of b.End5 in proximity to 4T1 cells with nuclear β4-integrin were counted.	
	β4-integrin	**4T1**	Mean Intensity	ImageJ	Measured the mean intensity circumscribed cell or cluster **(≥ 3 cells**).	One-way ANOVA
			Number of cells forming invadopodia		The number of 4T1 cells forming invadopodium (cytoplasmatic protrusions) was measured and divided by the total number of 4T1 cells per field. Data was shown as a percentage.	Kruskal-Wallis test
